# Two new species of *Chilocorus* Leach, 1815 from Laos (Coleoptera Coccinellidae Chilocorini)

**DOI:** 10.3897/BDJ.9.e72966

**Published:** 2021-12-02

**Authors:** Wenjing Li, Bingxu Chen, Chantharath Toulakhom, Xingmin Wang

**Affiliations:** 1 Guangdong Provincial Key Laboratory of High Technology for Plant Protection, Plant Protection Research Institute, Guangdong Academy of Agricultural Sciences, Guangzhou, China Guangdong Provincial Key Laboratory of High Technology for Plant Protection, Plant Protection Research Institute, Guangdong Academy of Agricultural Sciences Guangzhou China; 2 Key Laboratory of Bio-Pesticide Innovation and Application, Engineering Technology Research Center of Agricultural Pest Biocontrol, Guangdong Province; Engineering Research Center of Biological Control, Ministry of Education & Guangdong Province, South China Agricultural University, Guangzhou, China Key Laboratory of Bio-Pesticide Innovation and Application, Engineering Technology Research Center of Agricultural Pest Biocontrol, Guangdong Province; Engineering Research Center of Biological Control, Ministry of Education & Guangdong Province, South China Agricultural University Guangzhou China

**Keywords:** Coleoptera, Coccinellidae, *Chilocorus*, new species, Laos

## Abstract

**Background:**

*Chilocorus* Leach, 1815 the largest genus of Chilocorini, contains more than 80 species, mainly preying on Coccoidea. Many species of *Chilocorus* are economically important as they are widely used as biological control agents.

**New information:**

In this study, two new species of the genus *Chilocorus* Leach are described from Laos: *C.toulakhomianus* Li & Wang, sp. n. and *C.vientianicus* Li & Wang, sp. n. Diagnoses and detailed descriptions of the new species are given. Each species is illustrated in detail, including genitalia. Distribution maps are presented.

## Introduction

*Chilocorus* Leach, 1815 the largest genus of Chilocorini, contains 81 species in the recent world checklist (Li et al. 2018). However, morphological characters of the genus *Chilocorus* are relatively heterogeneous at species level. Chapin (1965) considered *Chilocorus* as a non-monophyletic genus. Miyatake (1970), who studied East-Asian species, subdivided *Chilocorus* into seven groups depending on the characters of the pronotal oblique line, prosternal process, prosternal hypomeral foveae, elytral epipleural foveae and elytral outer margin. Recently, the molecular and morphology-based phylogenetics of Chilocorini indicated *Chilocorus* as not a monophyletic group. Four other genera (*Phaenochilus* Weise, *Anisorcus* Crotch, *Egius* Mulsant and *Simmondsius* Ahmad & Ghani) were recovered as embedded in the clade of *Chilocorus*; meanwhile, these four genera were synonymised with *Chilocorus* (Li et al. 2020b).

Untill now, there is scarce literature to investigate and research Coccinellidae species in Laos. Prior to the prsent study, except for *C.politus* Mulsant, 1850 (Li et al. 2018), no other Chilocorini species was recorded in this country. This paper adds two new species to the world fauna of this genus from Laos. These two new species are extremely similar to *C.politus* in body shape and colouration.

## Materials and methods

Specimens, examined in this study, were collected in Laos. Type specimens of the new species are deposited at the Department of Entomology, South China Agriculture University (SCAU), Guangzhou.

External morphology was observed with a dissecting stereomicroscope (SteREO Discovery V20, Zeiss). Male and female genitalia were dissected, cleared in 10% solution of sodium hydroxide (NaOH) by boiling for several minutes and examined with an Olympus BX51 microscope. Photographs of the genitalia and other morphological characters were taken with digital cameras (AxioCam HRc and Coolsnap-Procf & CRI Micro*Color), attached to microscopes using AxioVision Rel. ver. 4.8 and Image-Pro Plus ver. 6.0. Images were cleaned up and laid out in plates with Adobe Photoshop CS ver. 8.0. Terminology follows Ślipiński (2007).

Abbreviations

TL = total length: length from apical margin of clypeus to apex of elytra; TW = total width: width across both elytra at widest part; TH = body height measured across the highest point of the elytra; HW = head width in a frontal view; PL = pronotal length: from middle of anterior margin to base of pronotum; PW = pronotal width at widest part; EL = elytral length: from apex to base including scutellum; EW = elytral width, equal to TW.

## Taxon treatments

### Chilocorus

Leach, 1815

49E810FC-B1D4-5426-9B37-6E51B06087D3

Chilocorus Leach, 1815: 116.
Chilocorus Coccinella cacti Linnaeus, 1767

#### Diagnosis

**Figure 1.**
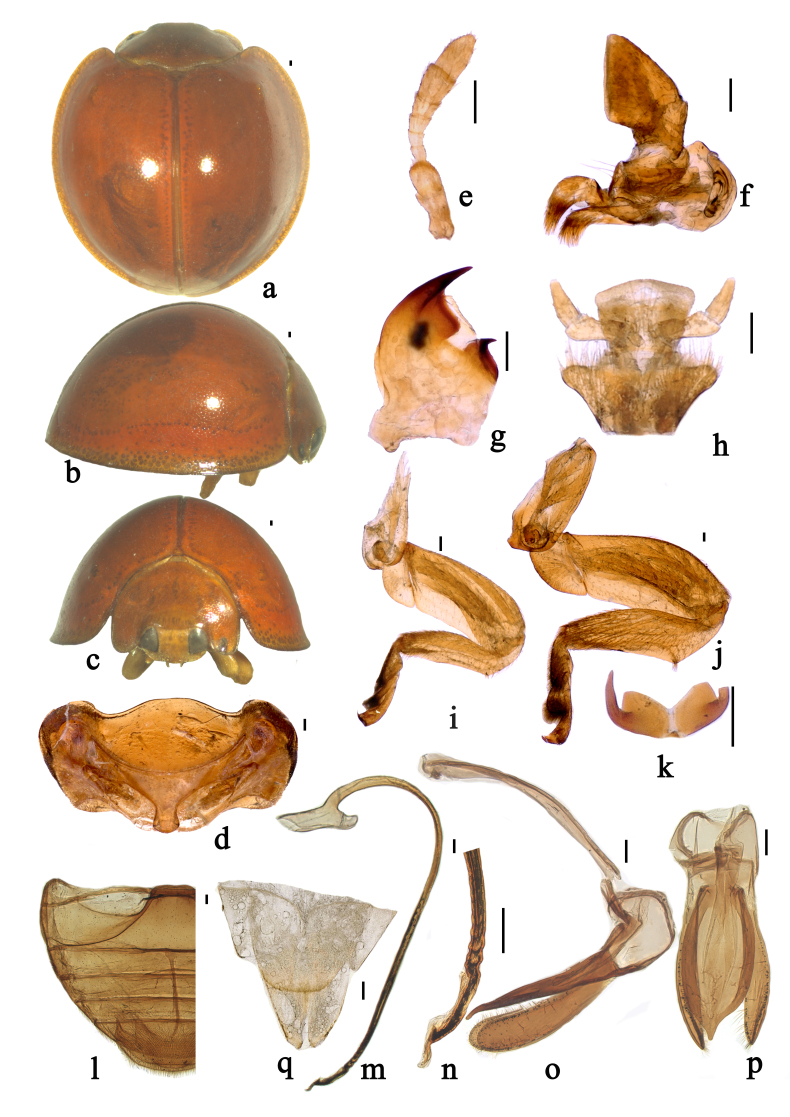
*Chilocorustoulakhomianus* Li & Wang, sp. n. **a** dorsal view; **b** lateral view; **c** frontal view; **d** prothorax; **e** antenna; **f** maxilla; **g** mandible; **h** labium; **i** front leg; **j** hind leg; **k** tarsal claw; **l** abdomen; **m** penis; **n** apex of penis; **o** tegmen, lateral view; **p** tegmen, ventral view; **q** ovipositor. Scale bars: 0.1 mm.

The genus *Chilocorus* can be distinguished from the other genera of the tribe Chilocorini by the following combination of characters: body with dorsum glabrous, rarely with pubescence; outer elytral margin slightly reflexed, without distinct bead; antenna stout, composed of 7 or 8 antennomeres (Fig. 1e); terminal maxillary palpomere elongate, from 1 to 2 times as long as basal width, with sides nearly parallel or moderately expanded to apex (Fig. 1f); prosternal process long, narrow and subparallel without carina (Fig. 1d); legs with stout femora, tibiae with a triangular tooth at basal 1/3, without tibial spurs (Fig. 1i–j); tarsal claws stout, with approximately rectangular basal tooth, about 1/2–2/3 as long as claw (Fig. 1k).

### Chilocorus toulakhomianus

Li & Wang sp. n.

3425B606-05DA-5F85-A495-0000FCA0D713

C6F187F8-0E6C-473D-861D-3D5BA2C58C27

#### Materials

- **Type status:** Holotype. **Occurrence:** recordNumber: No. 202107-01; recordedBy: Wenjing Li; individualCount: 1; sex: 1 male; lifeStage: adult; previousIdentifications: *Chilocoruspolitus*; **Taxon:** scientificName: *Chilocorustoulakhomianus* Li & Wang, sp. n.; **Location:** country: Laos; locality: Itou, Pakxong; georeferenceProtocol: label; **Identification:** identifiedBy: Wenjing Li; dateIdentified: 2021; **Event:** samplingProtocol: sweeping; eventDate: 06/12/2006
- **Type status:** Paratype. **Occurrence:** recordedBy: Wenjing Li; individualCount: 19; sex: 13 male, 6 female; lifeStage: adult; previousIdentifications: *Chilocoruspolitus*; **Taxon:** scientificName: *Chilocorustoulakhomianus* Li & Wang, sp. n.; **Location:** country: Laos; locality: Itou, Pakxong; georeferenceProtocol: label; **Identification:** identifiedBy: Wenjing Li; dateIdentified: 2021; **Event:** samplingProtocol: sweeping; eventDate: 06/12/2006
- **Type status:** Paratype. **Occurrence:** recordedBy: Wenjing Li; individualCount: 4; sex: 3 male, 1 female; lifeStage: adult; previousIdentifications: *Chilocoruspolitus*; **Taxon:** scientificName: *Chilocorustoulakhomianus* Li & Wang, sp. n.; **Location:** country: Laos; locality: Seleuy, Xam Nua; georeferenceProtocol: label; **Identification:** identifiedBy: Wenjing Li; dateIdentified: 2021; **Event:** samplingProtocol: sweeping; eventDate: 06/09/2007

#### Description

TL: 4.80–6.00 mm, TW: 4.45–5.28 mm, TH: 2.88–3.32 mm, TL/TW: 1.08–1.14, PL/PW: 0.57–0.61, EL/EW: 0.97–0.99.

Body roundish, strongly convex. Head, mouthparts and antenna brownish-red, sparsely covered with short, greyish pubescence. Pronotum, scutellum and elytra glabrous, brownish-red (Fig. 1a–c). Underside brownish-yellow, sparsely covered with short, greyish pubescence.

Head relatively large, 0.52–0.54 times pronotal width, punctures on frons large and densely distributed, 0.5–1.5 diameters apart, surface polished between punctures. Eyes approximately oval, densely faceted, interocular distance 0.45× as wide as head (Fig. 1c). Pronotum 0.50-0.52× as wide as elytra, pronotal punctures moderately large and moderately densely distributed, smaller than those on head, 1.5–3.0 diameters apart, surface polished between punctures. Punctures on elytra moderately large and sparsely distributed, 2.0–3.5 diameters apart, similar to those on pronotum. Prosternal process moderately broad, slightly expanded to apex. Abdominal postcoxal lines incomplete, reaching posterior margin of abdominal ventrite 1 and running along posterior margin, then almost reaching lateral margin. Posterior margin of male abdominal ventrite 5 broadly rounded and ventrite 6 slightly emarginate medially (Fig. 1l).

Male genitalia: penis slender, penis capsule with long outer and short inner arm, with spiral texture from apical 1/5 to apical 1/3, apex of penis truncate with membranous appendage (Fig. 1m–n). Tegmen stout, penis guide slightly narrow at base, gradually broadened up to basal 1/2, abruptly tapering from apical 1/4 to apex, slightly asymmetrical in ventral view; penis guide in lateral view, widest at base, gradually converging to apex; parameres nearly as long as the penis guide with dense short setae on the inner sides and distal end with a group of short setae in lateral view (Fig. 1o–p).

Female genitalia: coxites elongate, subtriangular, outer and inner margins almost straight, tapering to blunt apices (Fig. 1q).

#### Diagnosis

This species is very similar to *C.politus* and species *C.vientianicus*, sp. n. in the body size and colouration, but can be distinguished from them by penis with spiral texture from apical 1/5 to apical 1/3; penis guide widest at base, gradually converging to apex in lateral view. Furthermore, this species also can be distinguished from *C.vientianicus*, sp. n. by coxites with straight outer and inner margins. In *C.vientianicus*, sp. n., coxites outer margin is almost straight, but inner margin is distinctly concave near apical 1/3.

#### Etymology

The specific epithet is named after Toulakhom, the type specimen collector of this ladybird.

#### Distribution

Laos (Pakxong, Xam Nua) (Fig. 3).

### Chilocorus vientianicus

Li & Wang sp. n.

F6F39E35-8631-5282-9074-BEEAE640CCAE

061FD264-E148-4274-A6CC-2FE0F2E76B37

#### Materials

- **Type status:** Holotype. **Occurrence:** recordNumber: No. 202107-02; recordedBy: Wenjing Li; individualCount: 1; sex: 1 male; lifeStage: adult; previousIdentifications: *Chilocoruspolitus*; **Taxon:** scientificName: *Chilocorusvientianicus* Li & Wang, sp. n.; **Location:** country: Laos; locality: VangVieng, Vientiane; georeferenceProtocol: label; **Identification:** identifiedBy: Wenjing Li; dateIdentified: 2021; **Event:** samplingProtocol: sweeping; eventDate: 06/07/2006
- **Type status:** Paratype. **Occurrence:** recordedBy: Wenjing Li; individualCount: 8; sex: 4 male, 4 female; lifeStage: adult; previousIdentifications: *Chilocoruspolitus*; **Taxon:** scientificName: *Chilocorusvientianicus* Li & Wang, sp. n.; **Location:** country: Laos; locality: VangVieng, Vientiane; georeferenceProtocol: label; **Identification:** identifiedBy: Wenjing Li; dateIdentified: 2021; **Event:** samplingProtocol: sweeping; eventDate: 06/07/2006

#### Description

TL: 4.91–5.66 mm, TW: 4.58–5.35 mm, TH: 3.07–3.75 mm, TL/TW: 1.05–1.07, PL/PW: 0.54–0.57, EL/EW: 0.98–0.99.

**Figure 2.**
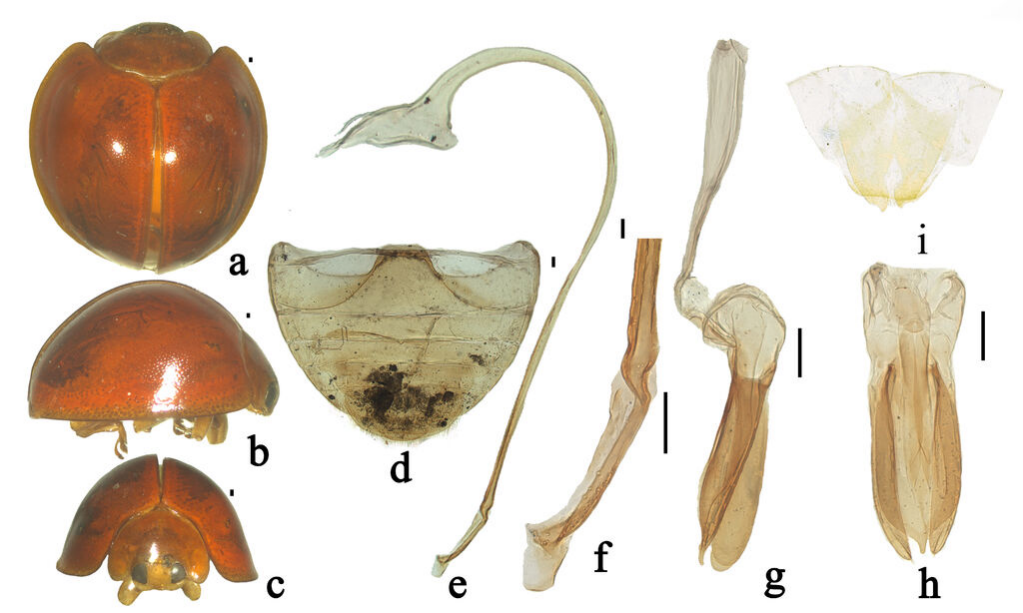
*Chilocorusvientianicus* Li & Wang, sp. n. **a**; dorsal view **b** lateral view; **c** frontal view; **d** abdomen; **e** penis; **f** apex of penis; **g** tegmen, lateral view; **h** tegmen, ventral view; **i** ovipositor. Scale bars: 0.1 mm.

Body roundish, strongly convex. Head, mouthparts and antenna brownish-red, sparsely covered with short, greyish pubescence. Pronotum, scutellum and elytra glabrous, brownish-red (Fig. 2a–c). Underside brownish-yellow, sparsely covered with short, greyish pubescence.

Head relatively large, 0.52–0.53 times pronotal width, punctures on frons large and densely distributed, 0.5–1.5 diameters apart, surface polished between punctures. Eyes approximately oval, densely faceted, interocular distance 0.47× as wide as head (Fig. 2c). Pronotum 0.53× as wide as elytra, pronotal punctures moderately large and moderately densely distributed, smaller than those on head, 1.5–2.5 diameters apart, surface polished between punctures. Punctures on elytra moderately large and sparsely distributed, 2.0–3.5 diameters apart, similar to those on pronotum. Prosternal process moderately broad, slightly expanded to apex. Abdominal postcoxal lines incomplete, reaching posterior margin of abdominal ventrite 1 and running along posterior margin, then almost reaching lateral margin. Posterior margin of male abdominal ventrite 5 broadly rounded and ventrite 6 slightly emarginate medially (Fig. 2d).

Male genitalia: penis slender, penis capsule with long outer and short inner arm, apex of penis truncate with membranous appendage (Fig. 2e–f). Tegmen stout, penis guide narrow at base, guide slightly narrow at base, gradually broadened up to basal 1/2, abruptly tapering from apical 1/4 to apex, approximately symmetrical in ventral view; penis guide in lateral view widest at base, parallel along basal 5/8, after that abruptly converging to apex; parameres nearly as long as the penis guide with dense short setae on the inner sides and distal end with a group of short setae in lateral view (Fig. 2g–h).

Female genitalia: coxites elongate, subtriangular, outer margin almost straight, inner margin distinctly concave near apical 1/3 (Fig. 2i).

#### Diagnosis

This species resembles *C.toulakhomianus*, sp. n. in the body size and colouration, but can be distinguished by the following characters: apex of penis truncate with membranous appendage, without spiral grain; penis guide widest at base, parallel along basal 5/8, after that abruptly converging to apex in lateral view; coxites elongate outer margin almost straight, inner margin distinctly concave near apical 1/3.

#### Etymology

The specific epithet is named after Vientiane, the type locality of this ladybird.

#### Distribution

**Figure 3.**
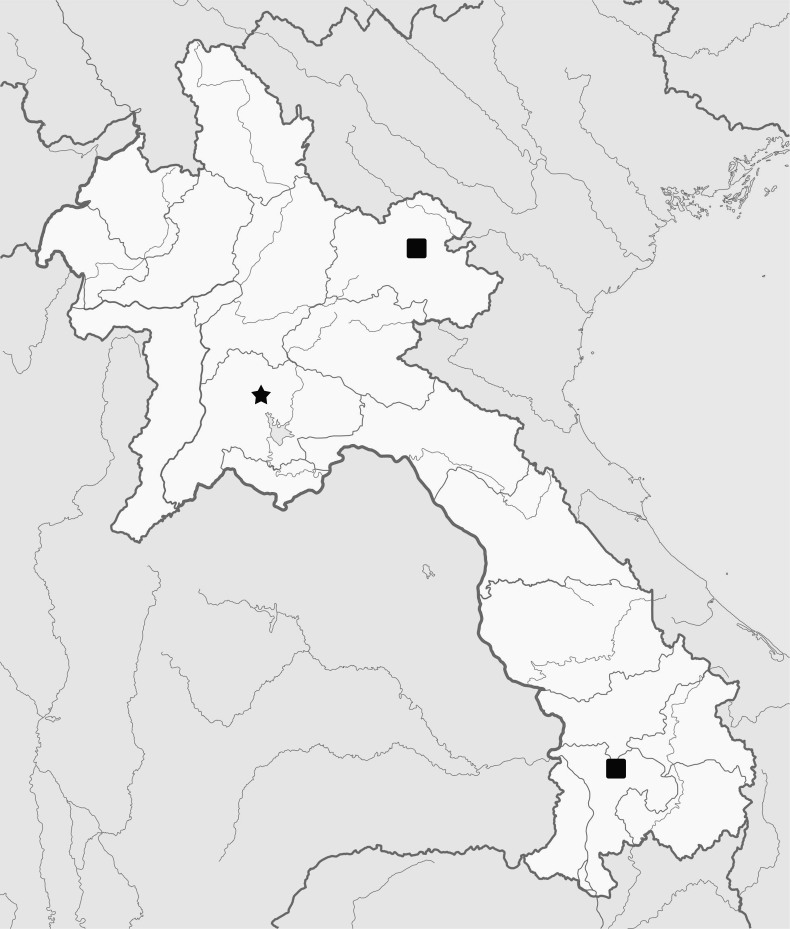
Distribution map. (■) *Chilocorustoulakhomianus* Li & Wang, sp. n. (★) *Chilocorusvientianicus* Li & Wang, sp. n.

Laos (Vientiane) (Fig. 3).

## Discussion

The species deversity of Chilocorini have been investigated in detail in many countries of East and Southeast Asia, such as China, Japan, Vietnam etc (Kamiya 1959; Miyatake 1970; Hoáng 1983; Li et al. 2020a). However, Chilocorini and even Coccinellidae in Laos are poorly taxonomically studied. In this paper, we describe two new species adding them to the world fauna of *Chilocorus* from Laos. As the largest genus of Chilocorini, the morphological characters of *Chilocorus* are relatively heterogeneous at species level. Compared with Asian fauna species, these two new species and their similar species *C.politus* can be easily distinguished by the following two characters: dorsum brownish-red without any spots; terminal maxillary palpomere moderately expanded to apex, the length nearly equal to the width.

## Supplementary Material

XML Treatment for Chilocorus

XML Treatment for Chilocorus toulakhomianus

XML Treatment for Chilocorus vientianicus
